# Diagnostic Accuracy of Radiomics in the Early Detection of Pancreatic Cancer: A Systematic Review and Qualitative Assessment Using the Methodological Radiomics Score (METRICS)

**DOI:** 10.3390/cancers17050803

**Published:** 2025-02-26

**Authors:** María Estefanía Renjifo-Correa, Salvatore Claudio Fanni, Luis A. Bustamante-Cristancho, Maria Emanuela Cuibari, Gayane Aghakhanyan, Lorenzo Faggioni, Emanuele Neri, Dania Cioni

**Affiliations:** 1 Radiology Department, Magnetic Resonance Service, Clínica de Occidente, Calle 18 Norte No. 5N 34, Cali 760045, Colombia; estefaniarenjifo@gmail.com; 2 Department of Translational Research, Academic Radiology, University of Pisa, Via Paradisa 2, 56124 Pisa, Italy; m.cuibari@studenti.unipi.it (M.E.C.); gayane.aghakhanyan@med.unipi.it (G.A.); lorenzo.faggioni@unipi.it (L.F.); emanuele.neri@unipi.it (E.N.); dania.cioni@unipi.it (D.C.); 3 Clínica Imbanaco, Cali 760042, Colombia; luis.bustamante@javerianacali.edu.co

**Keywords:** pancreatic cancer, pancreatic ductal adenocarcinoma, early diagnosis, radiomics, computed tomography, magnetic resonance imaging, METRICS

## Abstract

Background/Objectives: Pancreatic ductal adenocarcinoma (PDAC) is an aggressive and lethal malignancy with increasing incidence and low survival rate, primarily due to the late detection of the disease. Radiomics has demonstrated its utility in recognizing patterns and anomalies not perceptible to the human eye. This systematic literature review aims to assess the application of radiomics in the analysis of pancreatic parenchyma images to identify early indicators predictive of PDAC. Methods: A systematic search of original research papers was performed on three databases: PubMed, Embase, and Scopus. Two reviewers applied the inclusion and exclusion criteria, and one expert solved conflicts for selecting the articles. After extraction and analysis of the data, there was a quality assessment of these articles using the Methodological Radiomics Score (METRICS) tool. The METRICS assessment was carried out by two raters, and conflicts were solved by a third reviewer. Results: Ten articles for analysis were retrieved. CT scan was the diagnostic imaging used in all the articles. All the studies were retrospective and published between 2019 and 2024. The main objective of the articles was to generate radiomics-based machine learning models able to differentiate pancreatic tumors from healthy tissue. The reported diagnostic performance of the model chosen yielded very high results, with a diagnostic accuracy between 86.5% and 99.2%. Texture and shape features were the most frequently implemented. The METRICS scoring assessment demonstrated that three articles obtained a moderate quality, five a good quality, and, finally, two articles yielded excellent quality. The lack of external validation and available model, code, and data were the major limitations according to the qualitative assessment. Conclusions: There is high heterogeneity in the research question regarding radiomics and pancreatic cancer. The principal limitations of the studies were mainly due to the nature of the trials and the considerable heterogeneity of the radiomic features reported. Nonetheless, the work in this field is promising, and further studies are still required to adopt radiomics in the early detection of PDAC.

## Simple Summary

Pancreatic ductal adenocarcinoma is highly aggressive and usually deadly because is detected too late for effective treatment. Radiomics, a technique that uses patterns invisible to the human eye extracted from diagnostic imaging, could help identify early signs of this disease. The aim of this systematic review was to assess the application of radiomics to identify early indicators of pancreatic ductal adenocarcinoma. A review of ten studies explored how radiomics, particularly through CT, is being used to develop machine learning models to differentiate between healthy and cancerous pancreatic tissue. While promising, the studies showed mixed results due to heterogeneous findings and the methodological nature of the studies, highlighting the need for further research.

## 1. Introduction

The increasing incidence of pancreatic cancer, reaching about 1% annually in both men and women, has made it one of the five leading causes of cancer in the United States and European Union [1,2]. Moreover, according to the estimated cancer projections reported, it is expected to have further changes in incidence and the number of deaths [3,4].

Pancreatic cancer is an aggressive and lethal malignancy with a five-year survival rate of less than 10%, with pancreatic ductal adenocarcinoma (PDAC) being the most common and deadly histological subtype [1,5,6]. This dismal survival rate is primarily due to the late detection of the disease, which often occurs when the cancer has already reached advanced stages and is less responsive to effective treatments [7,8,9].

In this context, the early identification of PDAC is essential for improving treatment outcomes and long-term survival. Currently, diagnostic tools include imaging techniques such as computed tomography (CT) and magnetic resonance imaging (MRI) [8,10]. However, these techniques have significant limitations in terms of early detection and specificity, as they often struggle to differentiate between benign and malignant lesions in the early stages of the disease [8]. 

According to a retrospective review from Kang et al., amongst other factors, intrinsic tumor features such as small size (<2 cm), isoattenuation, or non-contour deforming may confound detection [11].

Radiomics may address the above-mentioned limitations by extensively extracting and analyzing quantitative features from medical images to create predictive models that link imaging characteristics to clinical outcomes [12,13]. Radiomics may assist in creating a function or mathematical model that classifies pancreatic lesions based on their anticipated outcomes using a combination of distinguishing features [14]. This technology has demonstrated its utility in recognizing patterns and anomalies not perceptible to the human eye through algorithms which learn from medical imaging data, identifying distinctive characteristics of precancerous or cancerous lesions, and, ultimately, providing physicians with more accurate and efficient diagnostic tools [15,16,17]. 

This study aims to systematically review the reported diagnostic accuracy of radiomics analysis of CT and MR images in the early detection of PDAC. By synthesizing data from various studies, this research seeks to contribute to the knowledge about the potential role of radiomics-based models in the early detection of pancreatic cancer.

## 2. Materials and Methods

A systematic literature review was conducted to identify original research papers pertaining to the application of radiomics in the analysis of pancreatic parenchyma images, aiming to identify early indicators predictive of PDAC. 

The literature search was performed on three databases, PubMed, Embase, and Scopus, according to the Preferred Reporting Items for Systematic Reviews and Meta-analyses (PRISMA) guidelines [18], and it was registered on PROSPERO (ID: ID=CRD42024572562). The following keywords, alone or in combination, were used: computed tomography OR magnetic resonance imaging AND radiomics OR artificial intelligence OR deep learning OR computer-assisted diagnosis OR machine learning AND early diagnosis AND pancreatic cancer. The last search was performed on July 16 2024.

The results were then exported to Rayyan, a cloud-based platform for screening citation data and for the automatic detection of duplicates [19]. Duplicates were then manually confirmed, and all the articles were initially screened by reviewing the title and the abstract by two reviewers (MERC and LABC), and conflicts were resolved by a third reviewer (SCF). Original research published in English and employing the radiomics methodology for the early diagnosis of PDAC on CT and MR images was considered eligible. Exclusion criteria included case studies with qualitative methodological approaches, systematic reviews, scoping reviews, other literature reviews, editorials, commentaries, letters to the editor, conference abstracts, proceedings, books, and book chapters.

From each study, the following data were extracted: study aim, the adopted imaging phase or sequence, segmentation strategy, software for radiomic features extraction, machine learning model, number of features extracted, and adherence to guidelines, such as the Image Biomarker Standardization Initiative guideline (IBSI), or checklist, such as the checklist for the evaluation of radiomics research (CLEAR) [20,21]. IBSI is an independent international framework that standardizes the extraction of radiomic features to ensure reproducibility and comparability across studies, while CLEAR is a guideline that helps assess the quality and transparency of radiomics research reporting [20,21]. The quality of the studies was assessed using the Methodological Radiomics Score (METRICS), a methodological scoring tool for assessing the quality of radiomics research, with a large international expert panel and a modified Delphi protocol (https://metricsscore.github.io/metrics/METRICS.html; accessed on 20 June 2024) [22]. This novel tool provides researchers with 30 items divided in 9 categories to assess, in a standardized manner, the methodological rigor of radiomic studies [22]. This scoring tool classified the articles into five categories representing the quality, i.e., very low quality (0 ≤ score < 20%), low quality (20 ≤ score < 40%), moderate quality (40 ≤ score < 60%), good quality (60 ≤ score < 80%), and excellent quality (80 ≤ score ≤ 100%) [22]. The qualitative analysis was carried out by two raters (MERC and MEC), and conflicts were resolved by a third reviewer (SCF).

## 3. Results

**Figure 1:**
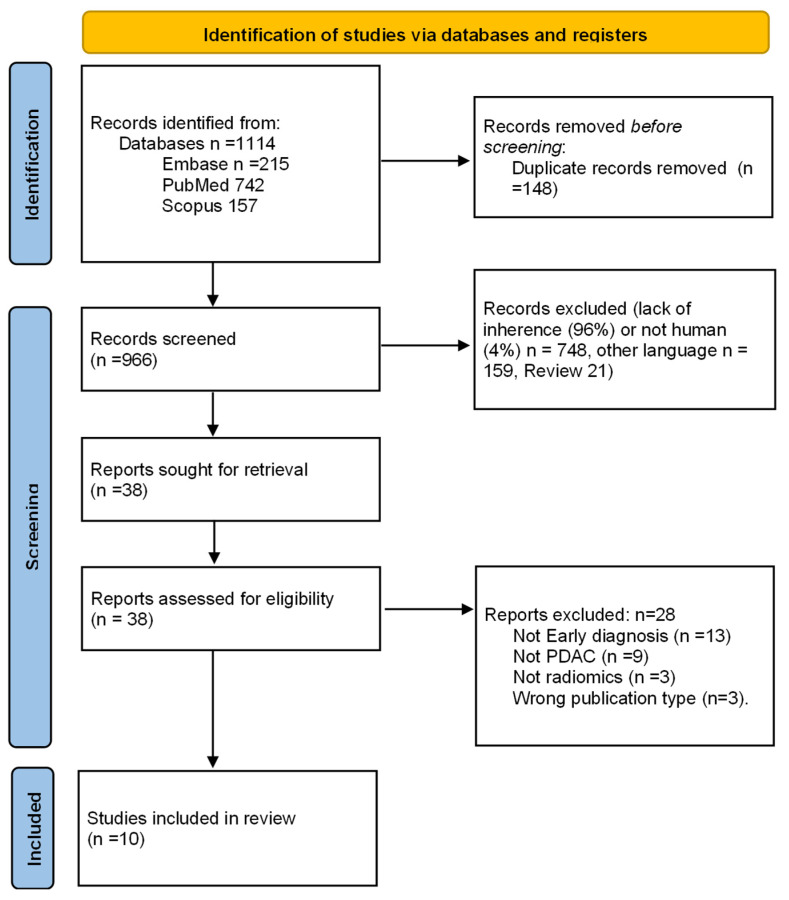
Study selection process flowchart according to the Preferred Reporting Items for Systematic Reviews and Meta-analyses (PRISMA) guidelines [18].

After deletion of duplicates, elimination of non-relevant articles, application of exclusion criteria, and resolution of 14 conflicts between the initial two reviews by a third reviewer, 10 studies were finally selected (Figure 1) [23,24,25,26,27,28,29,30,31,32].

The characteristics of the included articles are described in Table 1.

All of the studies were retrospective and published between 2019 and 2024 [23,24,25,26,27,28,29,30,31,32]. Analysis and radiomic feature extraction were carried out from CT scans, two of which were dual-energy CT (DECT) [25,27]. One of the studies using DECT also included MRI images for a multiparametric approach.

Eight out of the ten articles used one phase to extract the texture analysis features [23,25,26,27,28,32]. The selected phases in these articles were mainly venous or portal-venous phases [23,25,26,28,30,31,32]. The two studies that extracted their information from DECT decided to employ the arterial phase as their source [25,27]. Moreover, of these two, one also pulled out radiomic features from non-contrast images to compare accuracy [25].

In all but two of the articles [23,24,25,26,27,28,29,32], radiologists were involved in the segmentation process, with most of them being experienced. The conflicts or disagreements were resolved by a more experienced radiologist. The segmentation in the remaining study was carried out by trained researchers (MD students) under the supervision of an experienced radiation oncologist and a radiologist, respectively [30,31]. 

Most of the feature extraction was carried out using different versions of PyRadiomics [23,25,27,28,32], except for two. One used the binary mask and marching cubes algorithm [23], and the other used the analysis kit software (version V3.0.0.R, GE Healthcare) [29].

The number of features extracted was very large in all the articles, with the largest reported count to 4000 [24], and it was necessary to use different ways to eliminate the non-relevant and redundant features, selecting only the most accurate.

Five of the studies aimed at finding radiomic features that could differentiate PDAC patients from healthy controls [23,24,25,30,31]. The reported diagnostic performance of the model chosen yielded very high results, with a diagnostic accuracy between 86.5% [23] and 99.2% [24].

Two articles evaluated the pre-diagnostic CT scans [26,28]. The aim of Javed et al.’s study was to perform a risk prediction of pancreas subregions at high risk of developing PDAC. Using a model that differentiated the tumors on the diagnostic CT scans from healthy parenchyma, they carried out pre-diagnostic studies, obtaining a high accuracy (89.3%) in detecting the pancreas region with a high risk of developing a tumor [26]. Mukherjee et al. searched for pre-diagnostic CT scans (between 3 to 36 months before clinical diagnosis) and found a high accuracy (95%) of the ML model identifying PDAC in pre-diagnostic studies. Additionally, the performance of the radiologists was not only lower, but the inter-reader agreement was also only fair [28].

The purpose of Koch et al.’s study was to evaluate the diagnostic and predictive value of a multiparametric approach in terms of measuring the performance of the iodine concentration in the arterial phase, the ADC map on MRI, and texture features in order to differentiate inflamed tissue, tumors, and healthy pancreases. They reported a performance of 95% in distinguishing between malignant and inflamed pancreatic tissue for radiomics features, a performance of 95% for the iodine concentration, and a performance of 95% for DWI. Adding texture features to DWI and the iodine concentration increased the tumor detection rate to a diagnostic accuracy of 100% [25].

The objective of Ren et al.’s study was to evaluate the potential value of radiomics analysis in the differentiation of early-stage PDAC from late-stage PDAC using the most relevant and discriminative features, obtaining a with.7% accuracy in terms of distinguishing PDAC at an early stage (I–II) from at a late stage (III–IV) according to the AJCC staging system [10] on surgical specimens [29].

The majority of the articles followed radiomics guidelines or checklists, except for four of the included articles [24,26,29,31].

**Figure 2:**
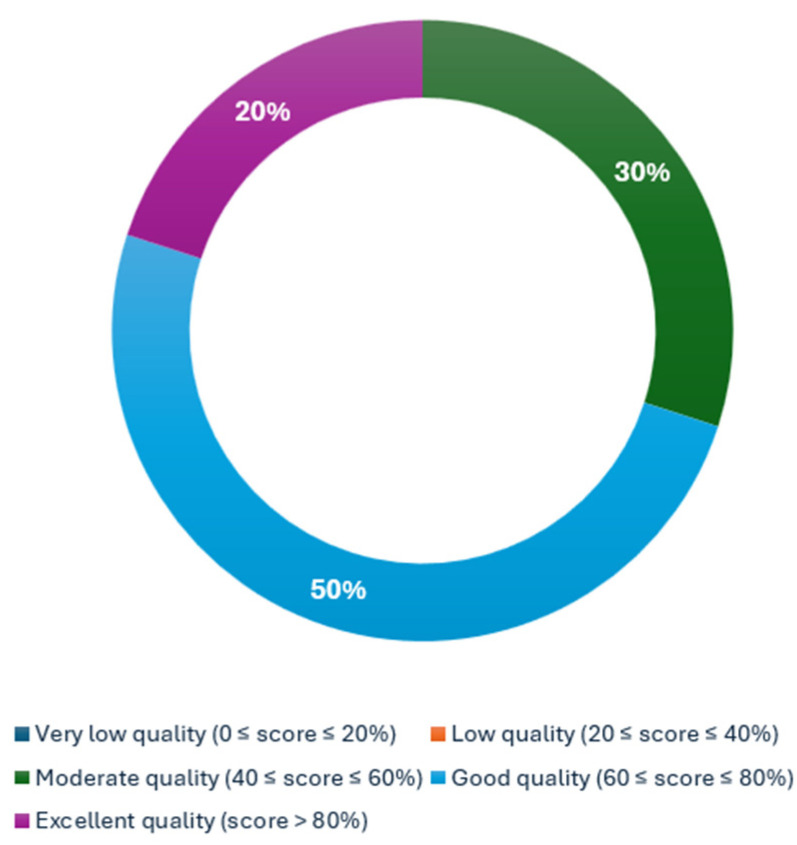
Distribution of METRICS quality categorization.

A qualitative assessment was conducted using METRICS. Among 336 ratings, 104 conflicts (31%) were resolved by a third reviewer. The METRICS scoring assessment demonstrated that three articles obtained a moderate quality category (Ren et al.: 57.1%, Chu et al.: 54.3%, and Mukherjee et al.: 52.2%) [29,31,32], five more obtained a good quality category (Chu et al.: 66.2%, Gotta et al.: 63.8%, Javed et al.: 69.3%, Koch et al.: 68.8%, and Wang et al.: 68.0%) [24,25,26,27,30], and, finally, two articles yielded an excellent quality category (Mukherjee et al.: 83.2% and Chen et al.: 86.2%) [23,28]. The proportion of the category assessment is reflected in Figure 2.

## 4. Discussion

As cancer is the second leading cause of death worldwide [2,33], efforts are always being made to improve its detection and efficient treatment, with the latter usually being dependent on the first one, hence the need to constantly search for more effective ways to diagnose cancer.

Medical imaging is an everyday tool in clinical practice, from screening and diagnosis to follow-up, with it also being present at treatment. Nonetheless, diagnostic imaging has its pitfalls and sources of misinterpretation, with some of them being due to the intrinsic nature of the organ or the disease [11,34]. Pancreatic cancer poses particular difficulty in diagnosis considering the size, localization, and complex function of the gland. PDAC diagnosis can be challenging because its presentation can be subtle and small, and it can have the same attenuation as the pancreas parenchyma [11,34,35].

Radiomics is a growing methodology that is gaining territory in clinical practice, that is used to extract and analyze features from diagnostic images, and that promises to help identify quantitative and reliable information that is easily missed by the human eye [11,34,35,36,37].

Owing to the high mortality rate of PDAC [2], which increases significantly with late diagnosis [9], we carried out a systematic literature review to assess studies on the early diagnosis of PDAC using radiomics. 

During our search, five of the studies aimed at finding radiomic features that could differentiate PDAC patients from healthy controls, i.e., those by Chen et al., Chu et al., Gotta et al., and Wang et al. [23,24,25,30,31]. The reported diagnostic performance of the models used yielded very high results, with a diagnostic accuracy between 86.5% and 99.2% [23,25,31]. Radiomics-based machine learning models may be accordingly adopted to augment the accuracy of diagnosing PDAC, targeting the heterogeneity of malignant parenchyma that might be missed by the naked eye [15,16]. Nonetheless, the reported average tumor sizes reported in these articles were usually detected by the radiologists [24,31]. This limitation highlights the need for original research investigating the stability of these models on smaller tumors, which are more challenging as they can be isodense to background tissue [35], with radiologists having to rely on secondary signs that might not be always present and, therefore, might cause false negatives.

To fulfill this need, some of the included articles investigated the potential role of radiomics-based models in reducing the incidence false negatives. On this subject, Chu et al. suggest that computer-aided diagnosis can be achieved using radiomics features without having to delineate both the pancreas and tumor [24]. The final goal of Chen et al.’s study was to propose radiomics-based models that could help to reduce incidences of missed PDAC, especially when CT images are used for general reasons and not only due to a suspicion of PDAC [23]. Gotta et al. reported a diagnostic accuracy of 98% in the arterial phase and 88% in the non-contrast phase and proposed fusing radiomics and DECT to detect the heterogeneity of tumors that can be challenging for the human eye to detect [25]. In the study by Wang et al., the robustness of radiomics features across the entire pancreas was tested against multiple sources of uncertainty, identifying 91 stable features that were the most important or “archetypal” for detecting early-stage cancerous changes [30]. This promises a computer-aided tool to improve PDAC detection, reduce false negatives, and enhance diagnostic accuracy by identifying critical features through machine learning approaches.

Conversely, Mukherjee et al. and Javed et al. demonstrated that ML models using radiomics features, which capture differences beyond human perception, can accurately identify PDAC in pre-diagnostic images [26,28,32]. Koch et al. used radiomic features, the iodine concentration from DECT, and DWI from MR images in a multiparametric manner, resulting in an improved ability to discriminate between malignant lesions and inflamed or normal pancreatic parenchyma, offering a higher diagnostic accuracy and better specificity than individual modalities. Given the increasing incidence of PDAC and the crucial role of its early surgery for survival [3,4,9,38], radiomics could provide a potential opportunity to screen high-risk patients, which are currently screened using MRI and endoscopic ultrasound, thereby helping to avoid mistreatment and time wastage [39,40,41,42,43].

Finally, Ren et al. emphasized the importance of properly discriminating early- from late-stage PDAC on its management. With this goal, they constructed a model with the nine most-predictive features to discriminate these two stages, achieving high positive and negative predictive values of 98.4% and 96.8%, respectively, hoping that a prospective and larger-scale cohort could validate the potential value of their findings [29], representing another possible window for further research.

However, due to the intrinsic physiological and anatomical nature of the gland [35], there are a high number of and is a vast heterogeneity in the mineable features of the pancreas and in pancreas diseases, making it difficult to pinpoint the replicable features on a large-scale model for screening processes. In our search, we found numerous features listed with variations that were considered the most important and reliable. Nevertheless, shape and texture features emerged as the primary factors in the majority of the studies.

The most common texture features reported included the grey-level co-occurrence matrix (GLCM) [23,24,25,27,28,29,32], which captures combinations of discretized intensities or grey levels of neighboring pixels or voxels along one of the image directions [44,45,46]; the grey-level dependence matrix (GLDM) [23,25,28,32], which reflects the coarseness of the overall texture, is rotationally invariant, and serves as an alternative to GLCM [44,45]; and, lastly, the grey-level size zone matrix (GLSZM) [25,28,32], which counts the number of contiguous zones (groups) of linked voxels that share the same discretized grey level. [45,46]. As for shape-based features, sphericity was reported in three of the trials, [24,30,31] where it was used to quantify the deviation from a representative spheroid [44,45,46].

Designing a large, multicenter, retrospective study using previously established technical minimum-standard CT data collected from already available images could help in identifying reliable and robust features for model development. These features could focus on discretizing grey levels, capturing texture coarseness, and identifying shapes that deviate from sphericity. The resulting models could then be tested in large-scale prospective studies to evaluate reproducibility, reliability, and generalizability across multiple centers.

We analyzed the methodological quality of the articles with the METRICS tool [22]. It is important to note that in nearly one-third of the ratings, a third experienced reviewer was required to resolve conflicts between the initial two ratings. This suggests that the evaluation may have limitations in terms of reproducibility. Further studies are needed to explore this issue.

This analysis showed that 80% of the articles achieved a rank of moderate-to-good quality, that is, five articles (50%) ranked as good [24,25,26,27,36], and three (30%) ranked as moderate [29,31,32]. Two articles achieved a rank of excellent quality, i.e., 25% [23,28].

This tool highlighted several limitations. Few of the studies thoroughly described their segmentation [23,25,27,28,32] and preprocessing techniques [23,28,29,30,32], which are crucial for accurate PDAC detection and the proper extraction of radiomics data. Additionally, only three studies used external validation [23,26,28], an important factor for ensuring applicability and reproducibility. Furthermore, only two articles referenced the use of AI guidelines or checklists [23,25] to guide their studies, which are essential for standardizing a level of quality and ensuring reliability. Moreover, there was insufficient sharing of code, data, and models, an issue addressed by the authors of METRICS [22], who emphasize open science practices to improve the generalizability of radiomics studies. Despite all these promises, the adoption of radiomics in clinical practice faces several ethical and technical challenges. Data privacy is a major concern, as radiomics relies on large datasets that often contain sensitive patient information, necessitating strict anonymization and secure data-sharing protocols [47]. Model interpretability is another critical issue; many radiomics models function as “black boxes,” making it difficult for clinicians to understand and trust their decision-making processes [48]. This lack of transparency can hinder clinical acceptance and integration into practice. Additionally, the potential impact on clinicians is significant—while radiomics tools can enhance decision-making, unreliable or non-interpretable models may lead to over-reliance on AI without sufficient clinical oversight [49]. Addressing these challenges requires robust validation, regulatory frameworks, and collaborative efforts between engineers, ethicists, and healthcare professionals.

## 5. Conclusions

Pancreatic ductal adenocarcinoma (PDAC) remains one of the deadliest cancers, with early diagnosis being critical to improving patient outcomes. Radiomics, which involves extracting and analyzing image features from diagnostic scans, has emerged as a promising tool for enhancing the early detection of PDAC. Our systematic review of the literature revealed that radiomic models based on CT scans achieved high diagnostic accuracy, ranging from 86.5% to 99.2%.

Despite the promising results, several limitations were identified in the studies reviewed. Important issues included inconsistent segmentation and preprocessing techniques, insufficient external validation, and a lack of adherence to AI guidelines—factors that impact the reproducibility and reliability of radiomic models. Moreover, most studies focused on CT scans with limited use of MRI, which is more commonly employed in PDAC screening.

Future research should address these limitations by establishing standardized imaging protocols, validating models across multiple centers, and exploring radiomics features from both CT and MRI. A large-scale, multicenter (potentially international), retrospective study utilizing previously established technical standards for CT imaging could help in identifying robust and reliable features for model development, particularly focusing on grey-level discretization, texture coarseness, and deviations from sphericity in pancreatic lesions. These features could then be incorporated into predictive models and tested in large prospective studies to assess their reproducibility, reliability, and generalizability across different centers.

Additionally, there is an opportunity to specifically target features from retrospective MRI data. While the sample size may be limited, integrating MRI-derived features could enhance detection accuracy and broaden the applicability of these technologies for screening high-risk patients.

Equally important is the need for larger-scale, multicenter, and prospective studies with standardized methodologies considering specific relevant and stable features to generate trustworthy models that can enhance the early detection of PDAC.

## Figures and Tables

**Table 1:** The characteristics of the articles included and the extracted data.

First Author. Year/Code	Title	Aim	Sample	Imaging Phase or Sequence	Segmentation	Extraction	Machine Learning Models	# of Features Extracted	Guidelines or Checklists	Metrics Score/Quality Category
Chen et al., 2021 [23]	Radiomic Features at CT Can Distinguish Pancreatic Cancer from Noncancerous Pancreas	To differentiate patients with PDAC and healthy controls	536 patients with PDAC and 579 healthy controls from Taiwan; 182 patients with PDAC and 82 healthy controls from the U.S.	CT -Portal venous	Two radiologist with 5 to 20 years of experience	PyRadiomics	XGBoost classifier	Not available	Image Biomarker Standardization Initiative guideline (IBSI)	86.2% Excellent
Chu et al., 2019 [24]	Utility of CT Radiomics Features in Differentiation of Pancreatic Ductal Adenocarcinoma From Normal Pancreatic Tissue	To determine the utility of radiomics features in differentiating CT cases of PDAC from normal pancreas	190 PDAC patients (97 men, 93 women) and 190 healthy controls	CT -Venous	Four researchers, approved by three radiologists with 5–30 years of experience	Binary mask to extract shape features and texture -first order- For computation of the shape marching-cubes algorithm, a filter was applied to capture isotropic heterogeneity	Random forest (RF)	478 Features	No	66.2% Good
Gotta et al., 2024 [25]	Unmasking pancreatic cancer: Advanced biomedical imaging for its detection in native versus arterial dual-energy computed tomography (DECT) scans	To investigate the potential of a machine learning classifier using DECT radiomics to differentiate between malignant pancreatic lesions and normal pancreas tissue	100 participants, of whom 60 patients received the final diagnosis of a histologically confirmed pancreatic cancer	DECT -Non-contrast and arterial	One radiologist with 5 years of experience in radiomics analysis	PyRadiomics	Gradient-Boosted Trees (GBTs)	107 Features	Checklist for the evaluation of radiomics research (CLEAR) and Image Biomarkers Standardization Initiative (IBSI)	63.8% Good
Javed et al., 2022 [26]	Risk prediction of pancreatic cancer using AI analysis of pancreatic subregions in computed tomography images	To perform a risk prediction of PDAC by automatically classifying the CT scans into healthy control (low-risk) and pre-diagnostic (high-risk) classes and specifying the subregion(s) likely to develop a tumor	108 CT scans from 72 subjects divided into internal (66 scans) and external (42 scans) datasets	CT -Venous	Two radiologists	Not reported	Naïve Bayes (NB) and recursive feature elimination	4000 total features	No	69.3% Good
Koch et al., 2023 [27]	Multiparametric detection and outcome prediction of pancreatic cancer involving dual-energy CT, diffusion-weighted MRI, and radiomics	To evaluate the diagnostic and predictive value of a multiparametric approach involving radiomics texture analysis, DECT iodine concentration, and diffusion-weighted MRI (DWI) in participants with histologically proven pancreatic cancer	143 participants: 83 with pancreatic cancer, 20 with pancreatitis, and 40 with no pancreatic pathologies	DECT -Arterial MRI -DWI	Two experienced radiologists (with 6 and 4 years of radiomics experience) and a third radiologist with 6 years of experience in radiomics for disagreements	PyRadiomics	Euclidean distance matrices, t-distributed stochastic neighbor embedding (t-SNE),Cox’s proportional hazards model, Intra-class correlation coefficients (ICC)	107 Features	Image Biomarkers Standardization Intiative (IBSI)	68.8% Good
Mukherjee et al., 2022 [28]	Radiomics-based Machine-learning Models Can Detect Pancreatic Cancer on Prediagnostic Computed Tomography Scans at a Substantial Lead Time Before Clinical Diagnosis	To detect PDAC at the prediagnostic stage (3–36 months before clinical diagnosis) using radiomics-based ML models and to compare performance against radiologists in a case–control study	Prediagnostic cohort (*n* = 155) control patients (*n* = 265)	CT -Portal venous	One of three radiologist with 2 to 4 years of experience	PyRadiomics	K-nearest neighbor (KNN), support vector machine (SVM), random forest (RF), and extreme gradient boosting (XGBoost)	88 features	Image Biomarkers Standardization Initiatve (IBSI)	83.2% Excelent
Ren et al., 2024 [29]	Computed tomography-based radiomics diagnostic approach for differential diagnosis between early- and late-stage pancreatic ductal adenocarcinoma	To evaluate the potential value of radiomics analysis in the differentiation of early-stage PDAC from late-stage PDAC	140 patients with pathologically proved PDAC	CT -Late arterial and portal venous	One experienced radiologist	Analysis Kit software GE Healthcare	Random forest (RF), leave-group-out cross-validation (LGOCV)	792 Features	No	57.1% Moderate
Wang et al., 2022 [30]	Compute Tomography Radiomics Analysis on Whole Pancreas Between Healthy Individual and Pancreatic Ductal Adenocarcinoma Patients: Uncertainty Analysis and Predictive Modeling	To establish a predictive model that can distinguish cancer patients from healthy individuals based on radiomics features analyzed from the whole pancreas of healthy individuals and pancreatic cancer patients	181 healthy controls and 85 cancer patients were included	CT -Venous	Two trained researchers (MD students) under supervision of an attending radiation oncologist with 18 years of experience	PyRadiomics	Random forest (RF), minimum redundancy maximum relevance (mRMR), leave-group-out cross-validation (LGOCV), univariate logistic regression	924 features	Image Biomarkers Standardization Intitiative (IBSI)	68.0% Good
Chu et al., 2020 [31]	Diagnostic performance of commercially available vs. in-house radiomics software in classification of CT images from patients with pancreatic ductal adenocarcinoma vs. healthy controls	To compare a commercially available and an in-house radiomics software in differentiating patients with PDAC and healthy controls	190 PDAC patients (97 men, 93 women) and 190 healthy controls	CT -Venous	Four researchers and approved by three radiologists with 5–30 years of experience	In-House software: binary mask and filtered images to extract features based on tumor intensity, shape, texture, and wavelet features. Commercial software: syngo.via Frontier, Siemens Healthineers	Random forest (RF), minimum redundancy maximum relevance (mRMR)	In-house software: 478 Features Commercial software: 854 Features	No	54.3% Moderate
Mukherjee et al., 2024 [32]	Assessing the robustness of a machine-learning model for early detection of pancreatic adenocarcinoma (PDA): evaluating resilience to variations in image acquisition and radiomics workflow using image perturbation methods	To evaluate the robustness of a radiomics-based support vector machine (SVM) model for the detection of visually occult PDA on pre-diagnostic CTs	155 pre-diagnostic CTs of 155 patients (90 men, 65 women) and 265 controls (140 men, 125 women)	CT-Venous	Three fellowship-trained radiologists	PyRadiomics	Support vector machine (SVM)	98 features	Image Biomarkers Standardization Initiative (IBSI)	52.2% Moderate

PDAC: pancreatic ductal adenocarcinoma, ML: machine learning, IMC: informational measure of correlation, NGTDM: neighboring grey tone difference matrix, GLCM: grey-level co-occurrence matrix, GLSZM: grey-level size zone matrix, GLDM: grey-level dependence matrix, HGLZE: high-grey-level zone emphasis.
